# Supplementary material for: Sodium Alginate/Chitosan Scaffolds for Cardiac Tissue Engineering: The Influence of Its Three-Dimensional Material Preparation and the Use of Gold Nanoparticles
Source: Polymers (Basel). 2022 Aug 9;14(16):3233. doi: 10.3390/polym14163233 (PMC9414310; doi:10.3390/polym14163233)
Supplement: Supplementary file 1 [file polymers-14-03233-s001.zip › polymers-1796826 supplementary final.pdf]

Supplementary Materials

Methods

Permeability analysis

Several approaches have been proposed to evaluate permeability of TE scaffolds. In this work, the descending head direct gravitational method was used. This pressure head changes because the pipe is used both to provide fluid flow through the scaffold and to measure the pressure head [34].

A custom device was constructed showed in Figure S1 to generate different levels of compressive strain on the scaffolds and to measure the variation in fluid height. The device has a standpipe that provides a head of water and at the same time allows to measure the volume of water that passes through the scaffold, a chamber that contains the scaffold and a reservoir for collecting water, as the method used by [35].

The initial height (H1= 29.3 cm) of fluid in the standpipe was recorded at time 1 (t1). While liquid permeated through the scaffold, the time (t2) required for the fluid head to drop from the upper to the lower level (H2= 27.5 cm) was recorded using a stopwatch.

The permeability (k) and hydraulic conductivity (K) of the scaffold were calculated on the basis of Darcy's law:

$$k = K\frac{\mu}{\rho g} \qquad (3)$$

$$K = \frac{a}{A}\frac{L}{t}\ln\frac{H_1}{H_2} \qquad (4)$$

where (μ) is the viscosity of the medium, (ρ) is the density of the medium, (g) is the gravity acceleration, (a) is the tube area, (A) is the cross-sectional area at the sample flow, (L) is the sample thickness (in this case of the scaffold) and (H_1) and (H_2) are the initial and final height of the tube through which the medium passes. We used a tube area of 28.27 mm^2, and L= 2 mm (scaffold thickness).

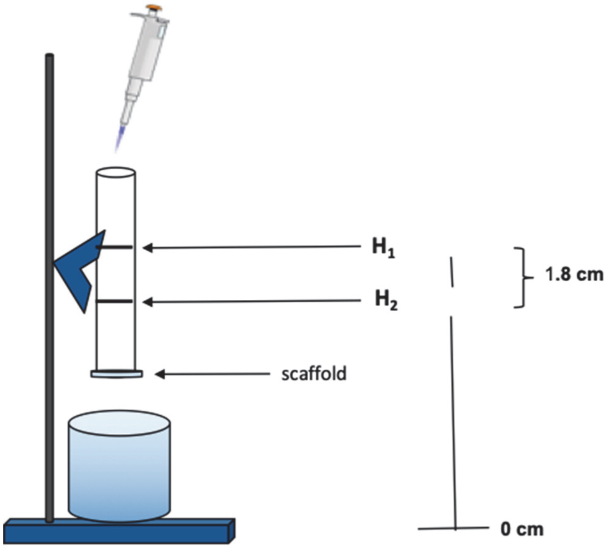

Figure S1. Custom device constructed to measure scaffolds permeability. It has a standpipe, a chamber that contains the scaffold and a reservoir for collecting water.

Results

Method 1 (without sonication) had the highest weight with respect to the other three fabrication methods ($p < 0.001$). Regarding thickness, the scaffolds generated by Method 2 (with sonication) were the thinnest, presenting significant changes with respect to Methods 1 and 3 ($p < 0.001$).

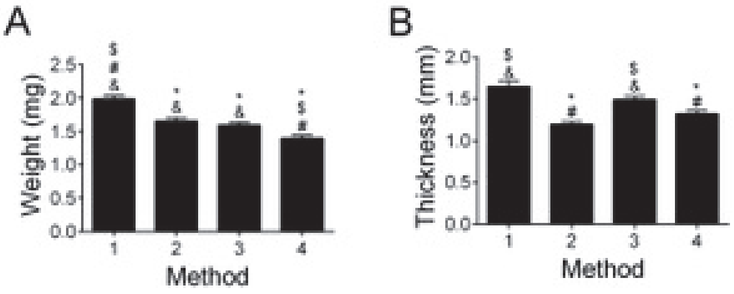

Figure S2. (**A**) Weight and (**B**) thickness of scaffolds according with fabrication method.

FTIR-ATR

Complete chemical characterization:

For sodium alginate (Figure 3A) the presence of the characteristic groups is confirmed [34]: OH (broad absorption band between 3650–3000 cm^{-1} with a peak at 3250 cm^{-1}, stretching), –CH (2930 cm^{-1}, stretching), COO– (1593 and 1407 cm^{-1}, asymmetric and symmetric stretching, respectively), C–O (1298 cm^{-1}, stretching), C–C and C–O (1082 and 1026 cm^{-1}),

and vibrational stretching bands due to mannuronic and guluronic acids (947, 889 and 816 cm^{-1}). Figure 3B shows the characteristic peaks of chitosan [35]: OH and NH overlap (3350 and 3290 cm^{-1}, stretching), −CH (2875 cm^{-1}, stretching), C=O (1644 cm^{-1}, stretching), NH (1581 cm^{-1}, bending), C–H (1375 cm^{-1}, bending), C–O–C (1150 and 1025 cm^{-1} of the glycosidic bond and in glucosamine, respectively, stretching). In the case of the scaffold with alginate/chitosan before crosslinking (Figure 3C), peaks can be observed that overlap due to the presence of both components (follow the dotted lines), and upon focusing on the peak at 1596 cm^{-1} it appears wider containing the overlap of the COO–, C=O (amide) and NH groups; in addition, the broad band between 3650 and 3000 cm^{-1} with respect to the –CH stretching group is at an intermediate intensity compared in the same way for the pure sodium alginate and chitosan components. The spectrum of the scaffold crosslinked with calcium gluconate by method 3 (Figure 3D), is similar to the spectrum of the scaffold before crosslinking (but an incorporation of OH and COO– due to the gluconate is present, and the following changes with respect to the –CH peak (~2900 cm^{-1}) of stretching can be observed: increase in the intensity of the peak at 1082 cm^{-1} indicated with a continuous line (C–O present in the calcium gluconate due to the OH present in its structure), decrease in the intensity of the peaks at 1589 cm^{-1} (COO– functionality) and in the broad band between 3650 and 3000 cm^{-1} (changes in the OH and NH groups). The IR spectra of the scaffolds crosslinked and with the incorporation of gold (AuNp) and gold plus alginate (AuNp+Alg) nanoparticles are presented in graphs 3E and 3F, respectively; they are similar to the scaffold before doping with gold nanoparticles.
